# Phenotypic characterization of liver tissue heterogeneity through a next-generation 3D single-cell atlas

**DOI:** 10.1038/s41598-024-53309-4

**Published:** 2024-02-03

**Authors:** Dilan Martínez-Torres, Valentina Maldonado, Cristian Pérez-Gallardo, Rodrigo Yañez, Valeria Candia, Yannis Kalaidzidis, Marino Zerial, Hernán Morales-Navarrete, Fabián Segovia-Miranda

**Affiliations:** 1 Department of Cell Biology, Faculty of Biological Sciences, Universidad de Concepción, Concepción, Chile; 2 Grupo de Procesos en Biología del Desarrollo (GDeP), Faculty of Biological Sciences, Universidad de Concepción, Concepción, Chile; 3 Max Planck Institute of Molecular Cell Biology and Genetics, Dresden, Germany; 4 Department of Systems Biology of Development, University of Konstanz, Konstanz, Germany; 5 Facultad de Ciencias Técnicas, Universidad Internacional Del Ecuador UIDE, Quito, Ecuador

**Keywords:** 3-D reconstruction, Multiphoton microscopy, Liver, Hepatic stellate cells, Hepatocytes, Image processing

## Abstract

Three-dimensional (3D) geometrical models are potent tools for quantifying complex tissue features and exploring structure–function relationships. However, these models are generally incomplete due to experimental limitations in acquiring multiple (> 4) fluorescent channels in thick tissue sections simultaneously. Indeed, predictive geometrical and functional models of the liver have been restricted to few tissue and cellular components, excluding important cellular populations such as hepatic stellate cells (HSCs) and Kupffer cells (KCs). Here, we combined deep-tissue immunostaining, multiphoton microscopy, deep-learning techniques, and 3D image processing to computationally expand the number of simultaneously reconstructed tissue structures. We then generated a spatial single-cell atlas of hepatic architecture (Hep3D), including all main tissue and cellular components at different stages of post-natal development in mice. We used Hep3D to quantitatively study 1) hepatic morphodynamics from early post-natal development to adulthood, and 2) the effect on the liver's overall structure when changing the hepatic environment after removing KCs. In addition to a complete description of bile canaliculi and sinusoidal network remodeling, our analysis uncovered unexpected spatiotemporal patterns of non-parenchymal cells and hepatocytes differing in size, number of nuclei, and DNA content. Surprisingly, we found that the specific depletion of KCs results in morphological changes in hepatocytes and HSCs. These findings reveal novel characteristics of liver heterogeneity and have important implications for both the structural organization of liver tissue and its function. Our next-gen 3D single-cell atlas is a powerful tool to understand liver tissue architecture, opening up avenues for in-depth investigations into tissue structure across both normal and pathological conditions.

## Introduction

The liver fulfils a wide range of functions, including metabolism, detoxification, protein synthesis, and production of biochemicals that aid digestion. These diverse functions rely on an intricate 3D tissue architecture where different cell types coexist and interact in a coordinated fashion, and thus, triangulating the exact spatial location of a cell is key to understanding its role. Macroscopically, the liver is composed of functional anatomical units called lobules, which include cellular and tissue structures located between the central vein (CV) and the portal triad (hepatic artery, portal vein (PV), and bile duct). The blood coming from the gut, pancreas, and spleen enters the liver via the PV and mixes with blood from the hepatic artery. Blood then flows toward the CV through a highly branched network of blood vessels called sinusoids, whereas bile flows through the bile canaliculi (BC) network in an antiparallel direction. The space between the central and the portal veins is filled predominantly by hepatocytes and non-parenchymal cells. Hepatocytes constitute the primary cell type at the core of liver function and are responsible for processing blood and secreting bile into the BC. They are “sandwiched” between the sinusoidal endothelial cells and share the apical surface with multiple neighboring hepatocytes to form a 3D BC network. This organization allows the hepatocytes to have numerous contacts with the sinusoid and BC networks to maximize the exchange of molecules^1^. Non-parenchymal cells also play important roles in liver function and growth regulation. They include liver sinusoidal endothelial cells, hepatic stellate cells (HSC), Kupffer cells (KC) and resident lymphocytes (dendritic, B, T, NK cells, etc.)^2^. HSCs are located in the space of Disse between sinusoids and hepatocytes, store Vitamin A, and secrete most of the extracellular matrix^3^. Indeed, activation of HSCs is a central driver of several liver diseases^3^. KCs are liver specific self-renewing resident macrophages located inside the sinusoidal capillary that play an important role in initiating hepatic immune responses and clearing circulating endotoxins^4^. Resident lymphocytes correspond to a heterogeneous and very small fraction of liver cells, however, they perform immunosurveillance participating in various liver diseases^5,6^. Increasing evidence suggests that hepatocytes, HSCs, and endothelial cells are in close contact, thereby, forming the so-called ‘hepatic niche’^7,8^. Therefore, it is not surprising that changes in the function of any cell integrated within the niche could eventually impact their neighborhood.

Classical histology has played a crucial role in understanding liver tissue structure. It is simple, versatile, and extremely accessible. However, this technique also presents several disadvantages, (1) it is not quantitative, (2) overlooks 3D information, (3) poorly distinguishes non-parenchymal cells, and (4) some tissue structures are not visible e.g., the BC network. Developments in tissue clearing, high-resolution fluorescence microscopy, and 3D image analysis have allowed 3D liver tissue reconstruction in the form of geometrical models^9,10^, to describe liver tissue architecture with unprecedented detail. Over the last few years, geometrical models have proven to be a game-changer and have illuminated basic principles of liver tissue organization. Some of the main findings include: (i) hepatocytes display a pronounced spatial zonation based on their ploidy^9^, (ii) the first predictive model of biliary fluid dynamics^11^, (iii) hepatocytes polarity exhibit liquid–crystal order^12^, (iv) liver regeneration after partial liver hepatectomy requires biomechanical growth control^13^, and (v) 3D reconstruction of human liver biopsies from non-alcoholic fatty liver disease patients show profound topological defects in the 3D BC network that lead to zonated micro-cholestasis^14^.

Till date, deep-tissue fluorescence microscopy is usually limited to a maximum of four fluorescent markers. Due to this restriction, 3D geometrical models have not been able to describe all the main cell types and tissue structures simultaneously, thus overlooking the organization of the hepatic niche. Simultaneous reconstruction of all important tissue networks (BC and sinusoids), cellular (hepatocytes, HSCs, and KCs) and sub-cellular components (nuclei) would require at least six different markers, making it impossible to observe all the structures of interest at once. Here, we combined deep tissue immunostaining, optical clearing, multiphoton microscopy, deep learning techniques, and 3D image processing to virtually expand the number of markers and generate a spatially resolved 3D single-cell atlas of the liver tissue, Hep3D. We used Hep3D to describe morphological changes established during early postnatal development and the structural role of KCs in liver tissue architecture. This atlas provides a powerful tool to quantitatively describe each cell type, its spatial organization, and its possible cross-interactions. Hep3D will help to identify (sub)structural characteristics of liver architecture, providing a quantitative tool to understand both liver biology and pathology with unprecedented detail.

## Results

### SeeDB optical clearing shows high compatibility with different staining modalities while preserving tissue morphology

To generate 3D geometrical liver tissue models, we first optimized our pipeline of deep-tissue imaging. Our standard pipeline involved several steps including, fixation, vibratome sectioning, staining, optical clearing, imaging at high resolution using multiphoton microscopy, and 3D reconstruction with the software Motion Tracking^9^. For staining specific structures, we used a CD13 antibody for BC, Flk-1 antibody for liver endothelial cells, and the small molecule dyes, DAPI (4,6-diamidino-2-phenylindole) and phalloidin, for nuclei and cell borders (actin mesh), respectively. Given the small size and densely packed arrangement of liver endothelial cells, discerning individual cells proved unfeasible. We therefore employed the Flk-1 marker to collectively identify all endothelial cells, effectively delineating the sinusoidal network as a whole. Optical clearing plays a pivotal role in ensuring the accurate generation of geometrical models by mitigating optical scattering within the tissue, thereby enabling deeper imaging. There are several optical clearing techniques, however, many of them are compatible with only a subset of markers or change the tissue morphology (e.g. tissue expansion)^15^. This can result in the extraction of inaccurate morphological parameters. Moreover, as most of these techniques were developed for the brain, there is scant information about their use in liver tissue^16^. To find the optical clearing method that best suited our requirements, liver slices were stained and optically cleared using different methods including, FOCM^17^, FRUIT^18^, RTF^19^, SeeDB2G^20^, SeeDB^21^, 3DISCO^22^, iDISCO^23^ and ECi^24^. We focused on well-stablished clearing methods that have been shown to preserve tissue morphology and tested them side-by-side in terms of staining compatibility and preservation of tissue morphology. Most of the clearing methods showed high compatibility with antibody staining, while their performance was variable with the small molecule dyes (Supplementary Fig. 1a). Even though the methods evaluated here showed different optical characteristics (Supplementary Fig. 1b), no major differences in tissue transparency were appreciable when 100 µm liver sections were compared (Supplementary Fig. 1a). It is likely that differences in transparency may be appreciable in thicker samples. Finally, we compared the effect of the optical clearing on tissue morphology both macroscopically (e.g. liver slice expansion) and microscopically (e.g. BC radius) (Supplementary Fig. 1c,d). FRUIT was the only method that resulted in tissue expansion both macro and microscopically. On the contrary, 3DISCO and iDISCO clearing methods caused tissue shrinkage. We found that SeeDB was the best clearing method for 100 µm tissue slices, showing high compatibility with different types of staining while maintaining tissue morphology.

### Virtual tissue labeling enables simultaneous 3D reconstruction of liver tissue components

**Figure 1:**
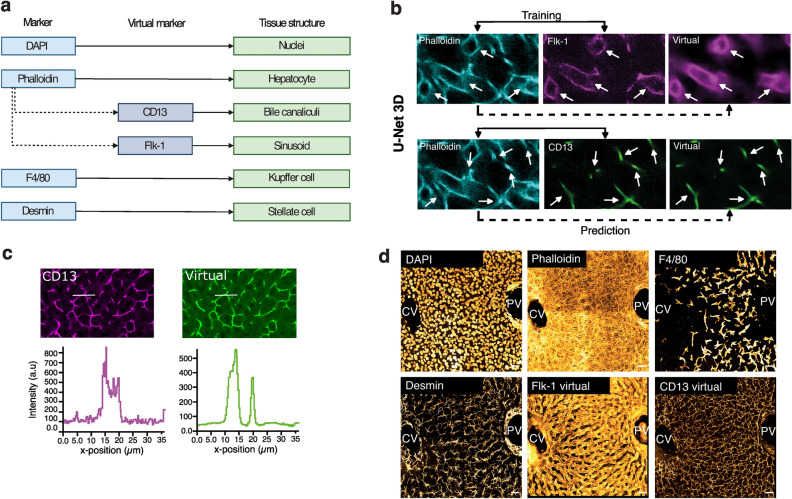
Deep tissue imaging and convolutional network allow the simultaneous observation of the main liver cell types and tissue structures. (**a**) Real and virtual markers used for each cell type and structure. (**b**) Images from 100 µm liver section stained with Phalloidin (input), Flk-1 (ground truth) and CD13 (ground truth). On the right, the predictions generated by the U-Net 3D model. White arrows indicate the sinusoidal and BC networks respectively. (**c**) Intensity profiles along the lines drawn on an example of CD13 marker (magenta) versus virtual CD13 (green). (**d**) Maximum projection of a 30 µm z-stack covering an entire CV-PV axis. Scale bar 30 µm. The training of the models involved approximately 36,000 (for BC) and 25,000 (for sinusoids) non-overlapping patches derived from six Z-stacks obtained from postnatal day 1, 16, and adult mice.

**Figure 2:**
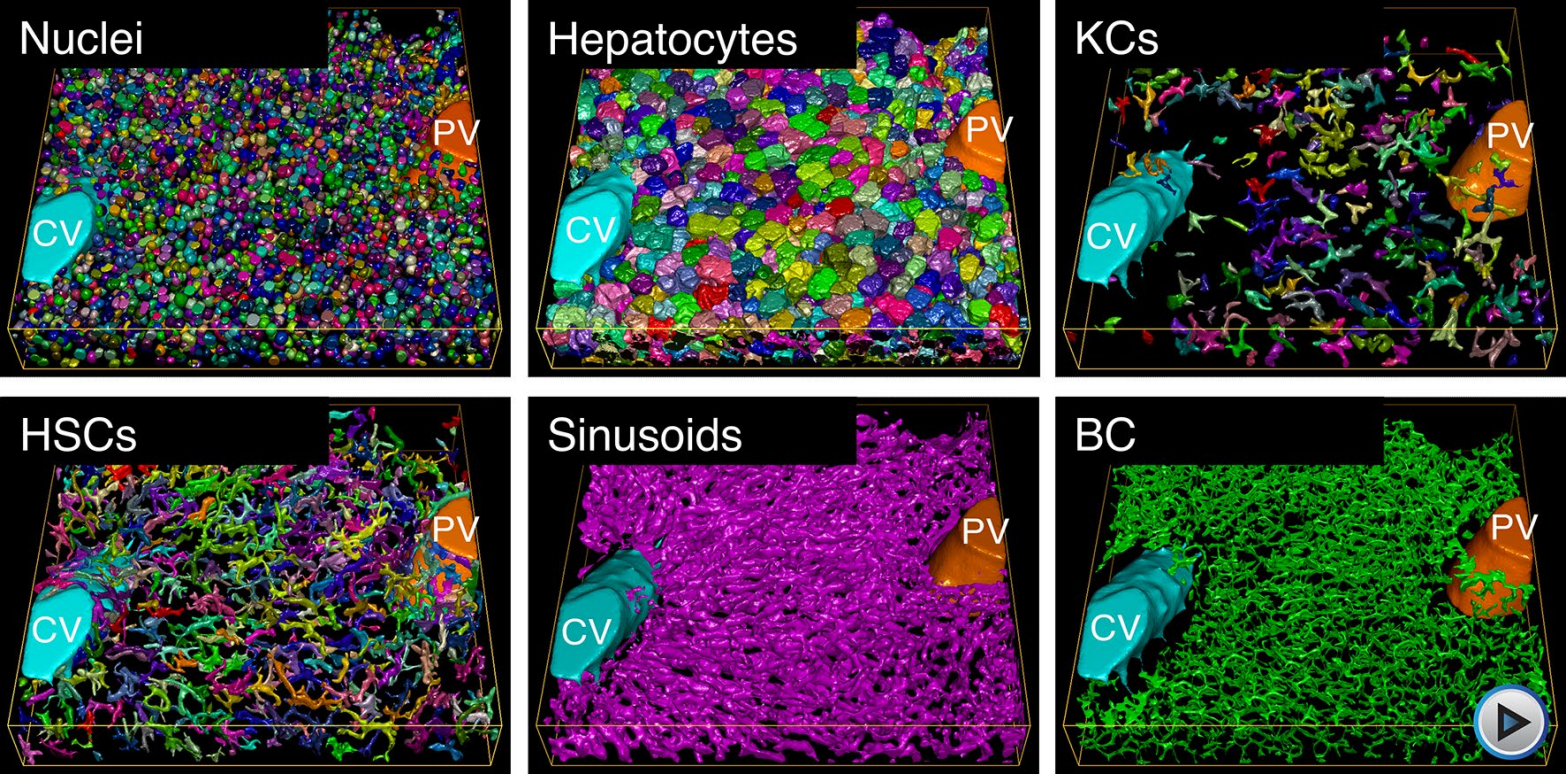
3D reconstruction of the main structures of the liver tissue. Central vein (light blue), portal vein (orange), nuclei (random colors), hepatocytes (random colors), KCs (random colors), HSCs (random colors), sinusoids (magenta) and bile canaliculus (green).

Deep-tissue fluorescence microscopy is usually limited to 4 markers to avoid bleed-through of the fluorescence emission^25,26^. However, in order to create a 3D geometrical tissue model that includes all the primary cell types and tissue components, it is necessary to image at least six distinct markers (Fig. 1a). Recently, deep learning techniques, particularly convolutional neural networks (CNNs) have been proven to be a power tool to predict virtual structures from membrane images^27,28^. We used CNNs to generate virtual 3D images of the BC and sinusoidal networks based on the phalloidin staining using CNNs (Fig. 1a–d and Supplementary Fig. 2). In particular, we expanded our previous 2D CNNs for the prediction of BC and sinusoids from phalloidin-stained images^27^ to a fully 3D model (Fig. 1b). The resulting predictions showed remarkable accuracy when comparing the virtual with the real BC and sinusoidal networks (Supplementary Fig. 2a–d). They showed a high signal-to-background ratio (Fig. 1c, Supplementary Fig. 2b,d) and their morphometric properties were very similar (Supplementary Fig. 2e,f). Following that, we replaced the antibodies originally employed to visualize BC and sinusoidal networks with the virtual markers. In their stead, we introduced antibodies targeting F4/80 and desmin to visualize KCs and HSCs, respectively (Fig. 1a,d). This approach allowed us to image and reconstruct all the main components of the liver tissue microarchitecture simultaneously, thus generating a multi-parametric 3D single-cell atlas of the liver which we named Hep3D (Fig. 2 and Supplementary Movie 1).

### 3D single-cell atlas reveals morpho-spatial differences in liver tissue architecture from early post-natal development to adulthood

As a demonstration of its potential, Hep3D was utilized first to examine the physiological structural changes that occur in the liver during early post-natal development. To this end, we focused on postnatal day 1 (P1), postnatal day 16 (P16), and adult mice. These temporal stages were selected as they encompass key phases in early postnatal development during which the liver undergoes several morphological changes. In P1 mice, the hepatocytes are mostly diploid and remnants of embryonic development such as hematopoietic cells can be detected^29,30^. In P16 mice, the liver tissue architecture is partially mature, as mice are weaned, triggering an increase in hepatocyte ploidy^31^. Adult mice displayed a mature and well-established liver tissue architecture.

Each 3D reconstruction comprises approximately 4000 cells for P1 and 2000 cells for the adult stage. Quantification of several morphological features including tissue (BC and sinusoidal networks) and cellular (hepatocytes, KCs, and HSCs) components was summarized in Supplementary Tables 1–3. Both BC and sinusoidal networks were fully connected at all stages. However, there were notable differences in their radii. While the BC radius decreased, the sinusoidal radius increased as the mice aged. The BC network corresponds to ~ 5% of tissue volume at all ages analyzed, however, the sinusoidal network increased from 14% in P1 to 25% in adults (Supplementary Table 1). Hepatocytes occupied around 58% of the tissue volume in all mouse stages analyzed. We observed that the morphology of hepatocytes became more heterogeneous as the mice aged, with variations in terms of nuclearity, ploidy, and cell volume. Hepatocyte cell volume changed from 2152 ± 160 µm^3^ in P1 mice to 4470 ± 525 µm^3^ in adults. The increase in cell volume was accompanied by a rise in the number of polyploid cells (41% in P1, 48% in P16 and 73% in adults), consistent with previous reports^32,33^. Other morphological properties, such as subdomains of the plasma membrane (apical, lateral and basal), and the number of neighbors, showed only minor changes (Supplementary Table 2).

HSCs were found to be highly elongated at all ages groups investigated. While the volume of individual HSCs increased with age, the nuclear volume decreased. Surprisingly, the number of HSCs reduced by ~ 42% from P1 to adult while they occupied a relatively constant proportion of the tissue (7–9%) (Supplementary Table 3). F4/80 positive cells were very elongated, and their number reduced as the mice aged (99,307 cells/mm^3^ in P1, 25,810 cells/mm^3^ in P16 and 13,345 cells/mm^3^ in adults). We also detected a significant reduction in the volume occupied by KCs during neonatal development (8.75% in P1, 6.95% in P16 and 3.57% in adults) (Supplementary Table 3). It is probable that a large fraction of the F4/80 positive cells in P1 represents not only KCs but also macrophages located within erythroblastic islets, which disappear from the liver about one week after birth in mice^34,35^.

**Figure 3:**
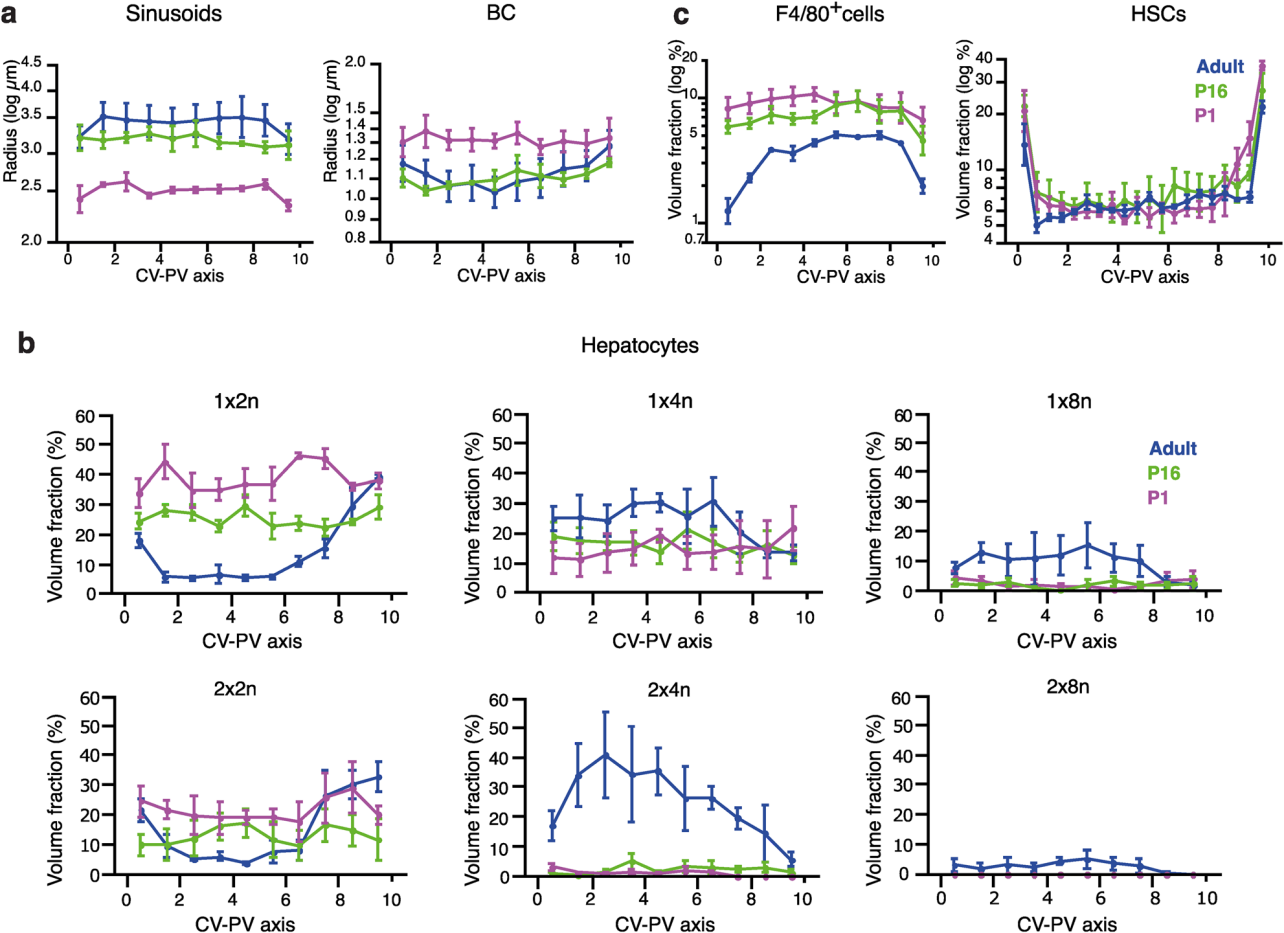
Morphological changes occurring during early postnatal development revealed by spatial analysis. (**a**) Spatial distribution of sinusoid and BC networks radius. (**b**) Quantification of the percentage of tissue volume occupied by hepatocytes with different ploidy along the CV–PV axis. (**c**) Quantification of the percentage of tissue volume occupied by F4/80 + cells and HSCs. In (**a**,**c**) the “y” axes are in log-scale. The measurements were taken from the CV to the PV, subdividing the space into 10 equal parts. P1 = 3 samples, P16 = 3 samples, Adults = 3 samples. Quantification represented by mean ± s.e.m.

Even though analyzing the cells in the liver as a population provided an extremely informative overview of the tissue, a detailed description of liver tissue structure has to take into account possible changes in morphology along the CV–PV axis^9,11,36,37^. Therefore, we computationally divided the CV–PV axis into ten equidistant regions and quantified the different morphological properties within each sub-region. While the spatial distribution of the sinusoidal radius appears homogeneous at all stages, the BC showed a modest increase towards the veins only in adults (Fig. 3a). Mono-nucleated diploid (1 × 2n) and bi-nucleated tetraploid (2 × 2n) hepatocytes were enriched toward the CV and PV, while hepatocytes with higher ploidies tend to be concentrated in the middle zone of adult livers (Fig. 3b), in agreement with previous reports^9,38^. Our data suggested that the spatial arrangement of hepatocytes according to their ploidy is an event that occurs after weaning (Fig. 3b). The spatial distribution of KCs and HSCs showed a clear anti-correlated pattern along the liver lobule for all ages. While KCs were enriched in the middle zone, HSCs were concentrated around the big veins (Fig. 3c). Our data supports previous findings where it has been shown that the average values, although important, hide important aspects of tissue architecture. In conclusion, Hep3D allows to capture detailed information about different liver cell types and their spatial organization.

### Hep3D unveils unexpected spatial interaction between HSCs and KCs

**Figure 4:**
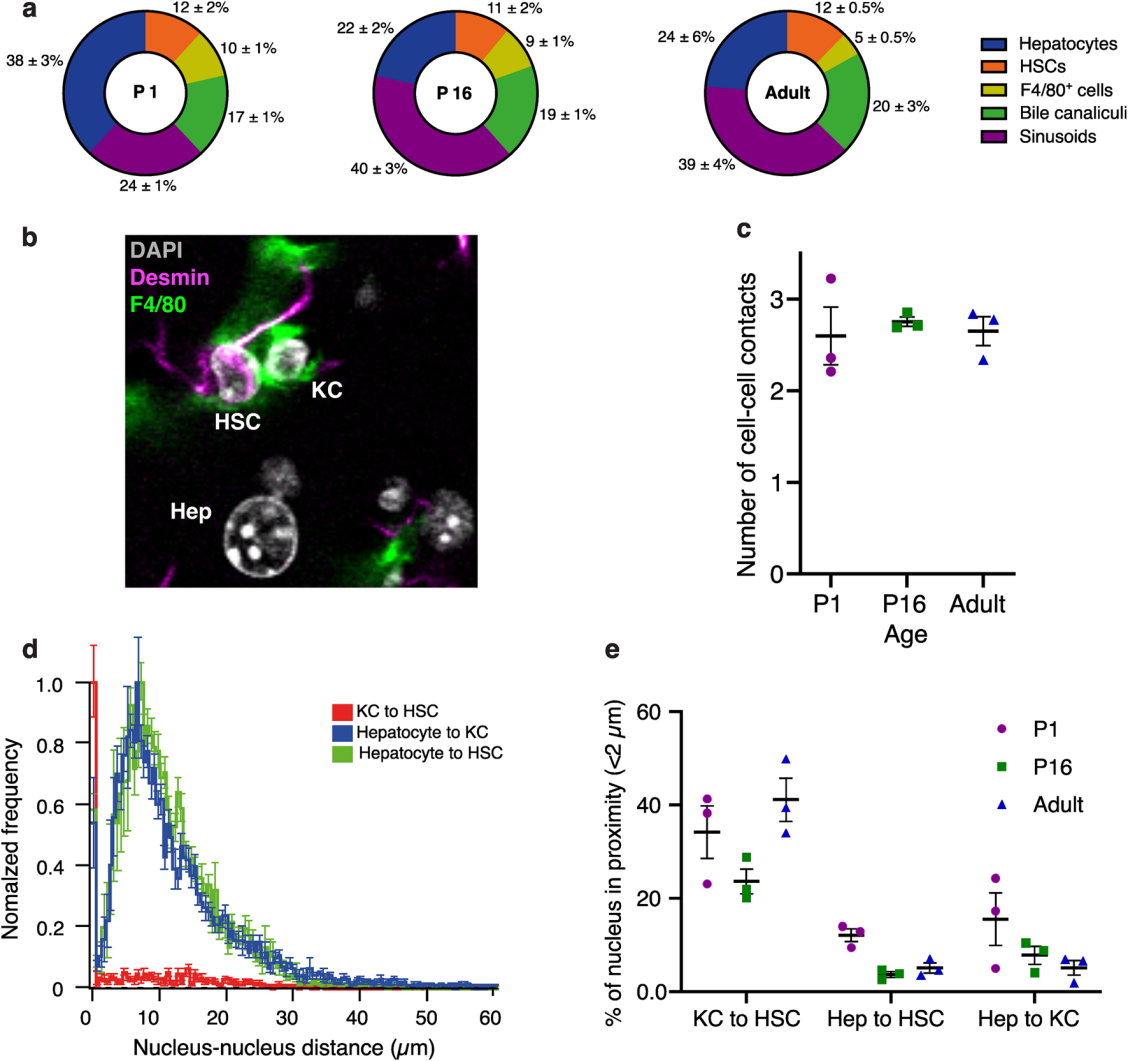
Interplay between cell types and tissue components. (**a**) Surface percentage of the hepatocytes in contact with the different structures (BC and sinusoids) and cells (HSCs, F4/80 + cells and other hepatocytes) present in the liver. (**b**) A maximum projection of 30 µm from the tissue showing the staining of desmin (red), F4/80 (green), Flk-1 (blue) and DAPI (magenta). (**c**) Average number of KC contact sites on the HSCs. (**d**) Normalized distribution of nucleus-nucleus distance between hepatocytes, KCs and HSCs. (**e**) Percentage of nuclei from different cell types which are closer than 2 µm. P1 = 3 samples, P16 = 3 samples, Adults = 3 samples. Quantification represented by mean ± s.e.m.

Having different cell types and tissue components simultaneously in our 3D reconstruction allowed us to do a systems level analysis of hepatic structure. First, we determined the percentage of hepatocyte surface engaged with other tissue structures. While certain areas of contact remained stable during mice maturation, such as HSCs and BC, interactions with other tissue constituents displayed changes; contact with sinusoids increased, whereas contact with neighboring hepatocytes and KCs decreased (Fig. 4a). Subsequently, we investigated the likelihood of physical proximity between HSCs and KCs, noting their close adjacency (Fig. 4b). This observation was quantitatively substantiated by assessing the number of cell–cell contact sites between HSCs and KCs. Our findings demonstrated that, on average, each KC exhibited 2–3 contact sites with HSCs. Interestingly, the count of contact sites remained constant as the mice matured (Fig. 4c). Additionally, we observed that the nuclei also appeared to be in proximity (Fig. 4b). To address this phenomenon, we quantified the distance between nuclei of diverse cell types (Fig. 4d). Examination of the distribution of inter-nuclear distances unveiled a remarkable closeness between KC nuclei and HSC nuclei (Fig. 4d). This observation was intriguing due to the elongated nature of KCs and HSCs, i.e. their nuclei could have been situated at a large distance from each other. Strikingly, we discovered that a substantial fraction (40 ± 5%) of KCs had nuclei closely positioned to HSC nuclei (inter-nuclear distance less than 2 µm) (Fig. 4e). In contrast, hepatocyte nuclei were, on average, separated from HSCs and KCs nuclei by at least 7–8 µm. This observation aligns with the spherical morphology of hepatocytes and the central location of their nuclei. These results propose that the proximity of HSC-KC nuclei is not a random occurrence, suggesting a robust direct interaction between KCs and HSCs, in line with previous reports^8,39^. To summarize, the results demonstrate that a thorough examination of 3D tissue morphology has the potential to unveil fundamental design principles governing the organization of liver tissue architecture.

### KC depletion leads to alterations in the structural arrangement of liver tissue

**Figure 5:**
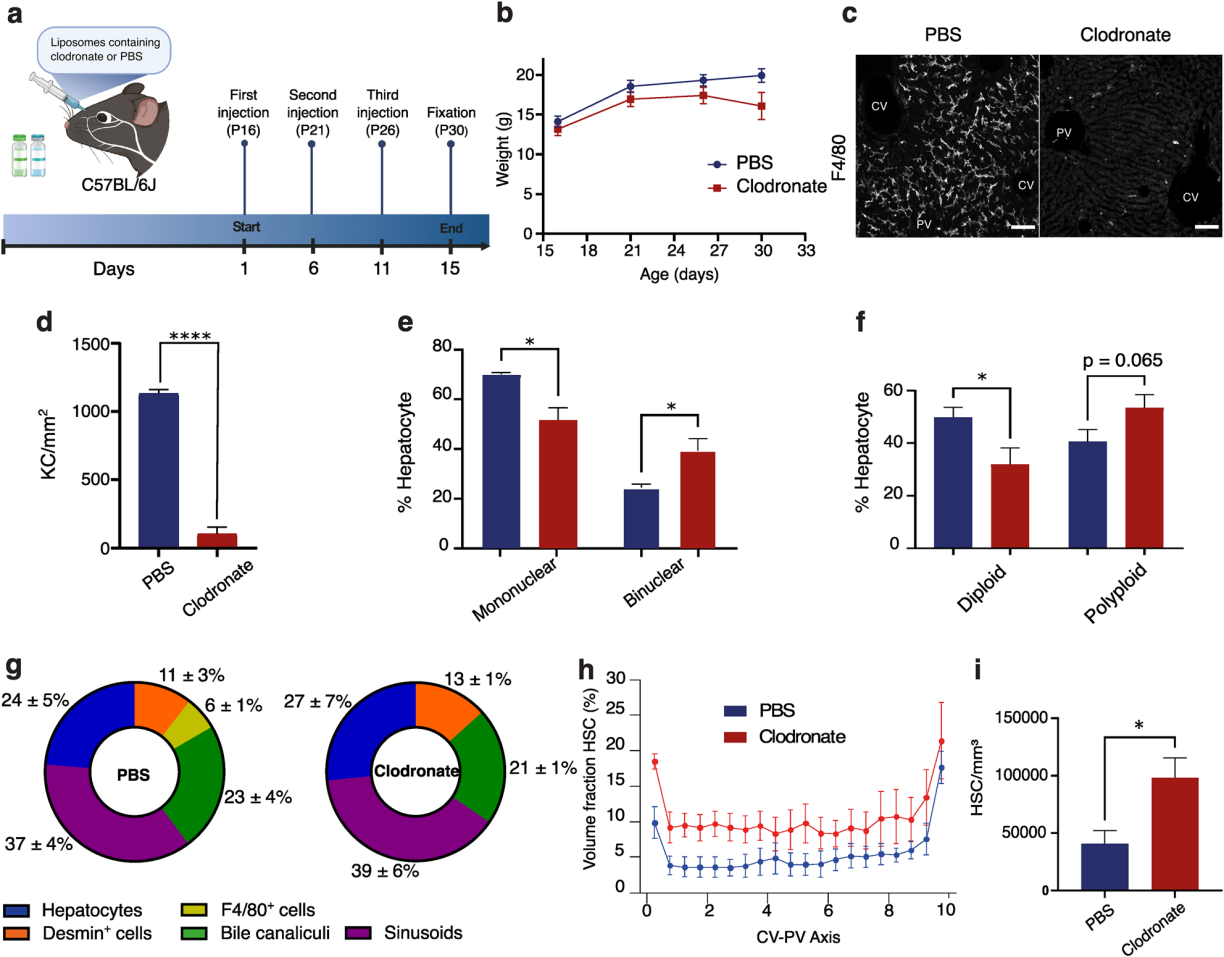
Structural defects of liver tissue architecture upon KC depletion. (**a**) Scheme showing the KC depletion experiment. Mice were injected with liposomes containing clodronate or PBS (control), every 5 days, starting at P16 until P30. (**b**) Mice were weighted every 3 days from the start to the end of the experiment. (**c**,**d**) 100 µm slices were stained with anti F4/80 and KC were quantified. Percentage of (**e**) mononuclear and binuclear, (**f**) diploid and polyploid and hepatocytes in PBS and clodronate conditions. (**g**) Percentage of hepatocyte surface contacting with other cell types and tissue structures. (**h**) Spatial distribution of HSCs volume fraction from PBS and clodronate. (**i**) Comparison of total number of HSCs/mm^3^ in PBS and clodronate. PBS = 3 samples, Clodronate = 3 samples. Quantification represented by mean ± s.e.m. One-tailed t-test (*P < 0.05, **P < 0.01, ***P < 0.001, ****P < 0.0001).

The different cell types in the liver exhibit a high degree of interconnection, forming a specialized microenvironment known as the hepatocyte's niche^8,40^. To investigate whether Hep3D has the capability to identify structural modifications resulting from the disruption of the hepatic niche, we conducted an experiment where we selectively removed the KCs population and assessed the consequences of this disturbance on the overall tissue architecture. In brief, KCs were depleted from the liver by intravenous injection of liposome-encapsulated clodronate^41,42^. To ensure the KC-depleted liver tissue had sufficient time to establish proper hepatic tissue architecture without causing undue distress to the mice, we first estimated the frequency of injections. Mice were retro-orbitally injected with clodronate and tissue samples were analyzed on days 1, 3, 5 and 7 post-injection (Supplementary Fig. 3). We observed that KCs started repopulating the liver on day 7, and therefore we injected the mice with clodronate every 5 days for long-term depletion experiments. Subsequently, mice were subjected to injections every 5 days from postnatal day 16 to day 30 (Fig. 5a,b). Clodronate treatment achieved a 91% reduction in the number of KCs/mm^2^ (Fig. 5c,d). At the structural level, the absence of KCs for 15 days (P16 to P30) did not cause detectable alterations on the BC and sinusoidal networks (Supplementary Table 4). Even though we only performed a short-term depletion, this duration proved sufficient to observe morphological changes in hepatocytes and HSCs. Specifically, hepatocytes retained most of their attributes except for nuclearity and ploidy, both of which showed an increase upon KC depletion (Supplementary Table 5 and Fig. 5e–g). This effect may be attributed to the previously described role of KCs on hepatocyte proliferation^40,43^, leading to potential alterations in hepatocyte ploidy^31^. Interestingly, the tissue volume occupied by HSCs increased dramatically upon KC depletion (Fig. 5h and Supplementary Table 6). This expansion was evident across the entire liver lobule (Fig. 5h) and was accompanied by a substantial rise in HSC numbers (Fig. 5i). In order to eliminate the potential influence of activated HSCs on the observed structural changes resulting from KC depletion, we conducted α-smooth muscle actin (α-SMA) staining. Our findings demonstrated no discernible distinctions between mice that received control liposomes and those that were administered clodronate liposomes (Supplementary Fig. 4), where α-SMA was only located around CV and PV, as expected. In concordance with previous reports, our morphological analysis of liver tissue support the existence of a direct crosstalk between KCs and HSCs^44,45^. In summary, our findings indicate that Hep3D can detect even subtle alterations within the hepatic niche.

## Discussion

Geometrical models have proven to be powerful and versatile tools to quantitatively describe the morphology of liver micro-architecture (BC and sinusoids)^11,46,47^ and its cells (hepatocytes, KCs, and HSCs)^9,48^. Unfortunately, it has not been possible to image and reconstruct all these structures simultaneously due to technological limitations. Numerous protocols have been devised to enable the simultaneous imaging of various markers, aiming to provide comprehensive insights into complex biological systems^49–51^. However, a significant proportion of these protocols heavily depend on the use of chemical reagents to detach antibodies, thereby allowing the sequential staining of different markers. While these methods hold promise for multiplex imaging, a critical concern arises regarding their potential impact on tissue morphology and integrity. Furthermore, many of these protocols are primarily applicable to thin tissue sections, limiting their capacity to capture a holistic view of 3D tissue structures. In this study, we showed that a combination of deep-tissue imaging, deep learning and traditional 3D image analysis techniques enables the 3D reconstruction of the main structural components of liver tissue architecture simultaneously. Our 3D single-cell digital atlas of the liver, Hep3D, includes hepatocytes, HSCs, KCs (both nuclei and cell surfaces), BC, and sinusoidal networks. Hep3D has facilitated the extraction of spatiotemporal patterns in morphological tissue attributes across distinct postnatal developmental stages. As a proof of concept, we described how liver tissue structure changes during the physiological transition from post-natal early development to adulthood, shedding light on the pivotal role of KCs in this progression. In alignment with prior research, our findings unveiled an elevation in hepatocyte ploidy with aging, coupled with distinct regional distributions within the liver lobule^38,52,53^. Intriguingly, an unexpected anticorrelated spatial arrangement of HSCs and KCs emerged. The intercellular interactions between these populations became conspicuously apparent upon KC depletion, significantly influencing the abundance and morphology of HSCs. Hep3D provides a holistic yet in-depth overview of liver tissue organization, with the potential for detecting even subtle changes in liver microarchitecture.

The combination of experimental data with computational models of tissues has proven successful in revealing how different tissues and organs function^11,54^. Volumetric imaging and optical clearing techniques play crucial roles in this process^55^. In this scenario, tissue preservation is paramount, as data extracted from the 3D reconstructions is not only used to get a quantitative understanding of tissue morphology but also, as an input for mathematical models. In this study, we evaluated different optical clearing techniques to achieve volumetric imaging and tissue preservation. SeeDB showed the highest compatibility with our staining method and the lowest impact on tissue morphology. Indeed, 3D reconstruction of BC showed morphometric parameters indistinguishable from samples without optical clearing. Unfortunately, SeeDB is not the best technique in terms of transparency so if a specific application requires the user to image deeper into the tissue, the development of new methodologies or the use of new microscopy techniques (i.e. 2-photon and light-sheet microscopy) will be necessary^55^.

Another important component of the pipeline is accurate 3D reconstruction of the tissue. Even though they are powerful and versatile tools, their efficient generation still poses some difficulties. The main challenges that we faced were related to the accurate segmentation of densely packed irregular shaped nuclei, and the segmentation of cells with complex shapes e.g. HSCs. The field of artificial intelligence is revolutionizing bioimage analysis and is actively providing new tools to overcome these problems^56^. In the future, it will be interesting to explore methods such as Stardist^57^, Cellpose^58^ and Plantseg^59^ to improve our 3D reconstructions. Furthermore, the integration of other deep learning techniques holds promise for advancing Hep3D. This includes the potential inclusion of less abundant cell types (e.g., resident lymphocytes) and the differentiation between activation states (e.g., M1 and M2 KCs), thereby broadening the scope and depth of our atlas.

Recently, a lot of effort has been devoted to understanding tissues at the cellular level^60^, even integrating spatial information^61^, e.g. single-cell spatial transcriptomics. While these technologies are a breakthrough in cell biology at the tissue scale, they usually overlook tissue morphology and niche organization, and consequently, their role in tissue biology and pathology. One example is our finding about the spatially anti-correlated organization of the KC and HSCs, wherein regions with a high density of HSCs showed a lack of KCs, and vice versa. Strikingly, in regions where these cell types coexist, we noted an intimate spatial proximity of their nuclei (less than 2 µm). In alignment with prior studies, our morphological analysis of liver tissue supports a direct interaction between KCs and HSCs^8,40^. While the specific mechanisms underlying these interactions require further investigation, our findings suggest that the absence of KCs has profound effects on HSCs. These insights contribute to the growing body of knowledge surrounding the complex interplay between different cell types within the liver microenvironment, shedding light on potential avenues for therapeutic interventions. Hep3D will provide a key tool to survey the principles of liver tissue organization under diverse conditions.

This versatile tool also has vast potential for advancing our understanding of liver diseases. By conducting 3D reconstructions of human liver biopsies from patients with non-alcoholic fatty liver disease, researchers were able to discover a set of cellular and tissue parameters correlated with disease progression^14^. Remarkably, the 3D analysis of a subset of liver tissue components was enough to discover profound defects in the BC network that were otherwise unnoticed^14^. Furthermore, the integration of the morphometric analysis of the BC network with mathematical modeling allowed to create personalized biliary fluid dynamic simulations^14^. In this regard, Hep3D stands to facilitate a multiparametric and quantitative examination of the interplay among essential cellular and tissue components, offering a promising avenue for investigating liver pathologies like the progression from steatosis to hepatocellular carcinoma. Furthermore, its potential extends to emerging disciplines such as phenomics, which focuses on achieving in-depth understanding of phenotypic attributes through the utilization of high-throughput and high-dimensional phenotyping methodologies^62,63^. Hep3D is a valuable and versatile tool for future investigations on the quantitative description of liver tissue microarchitecture in a wide variety of physiological and pathological conditions.

## Methods

### Animals

Postnatal day 1, 16, and adult C57BL/6J mice were obtained from the animal facility (Centro Regional de Estudios Avanzados para la Vida (CREAV)) at the Universidad de Concepción. The animals were maintained in strict pathogen-free conditions and received ad libitum feeding. All procedures performed were approved by the vice rectory of ethics and biosecurity committee from the investigation and development of Universidad de Concepción (ethics approval number CEBB 635–2020). In addition, this study was conducted according with the ARRIVE guidelines, and all methods were performed in accordance with the relevant guidelines and regulations.

### Sample collection and immunostaining

Mice livers were fixed through intracardiac perfusion with 4% paraformaldehyde in PBS containing 0.1% Tween-20 and post-fixed overnight with the same solution at room temperature. In the case of P1 mice, the livers were collected and fixed by immersion in 4% paraformaldehyde in PBS containing 0.1% Tween-20 over 5 days at room temperature. 100 µm thick liver sections were obtained with a vibratome. Immunolabeling (Supplementary Table 7) and optical clearing were performed as described previously^12^.

### Evaluation of optical clearing methods

Once the immunolabeling was performed, liver tissue sections were cleared by different methods. The protocols used included SeeDB2G^20^, SeeDB^12,21^, FRUIT^18^, FOCM^17^ , 3DISCO^22^, iDISCO^23^ and ECi^24^. Samples in PBS (uncleared) were used as a control. To estimate the macroscopic changes in the size of the tissue, the overall area was measured by drawing the contour of the liver slice before and after the clearing using the software Fiji^64^. Additionally, to measure microscopic changes, BC were stained and segmented and their radius was quantified using the software Motion Tracking^9^. The difference in both areas and the BC radius were compared to the control condition to determine if the tissue shrunk or expanded.

### Imaging

Liver samples were imaged (0.3 µm voxel size) in an inverted multiphoton laser-scanning microscope (Zeiss LSM 780) using a 40 × 1.2 numerical aperture multi-immersion objective (Zeiss). DAPI was excited at 780 nm using a Chameleon Ti–Sapphire 2-photon laser. Alexa Fluor 488, 555 and 647 were excited with 488, 561 and 633 laser lines and detected with Gallium arsenide phosphide (GaAsp) detectors. One CV-PV axis from a random lobe was imaged per mouse (~ 500 × 250 × 100 µm).

### Image pre-processing

The different components of liver tissues (BC, sinusoids, nuclei, HSCs, KCs and Hepatocytes) were reconstructed from high-resolution (voxel size 0.3 × 0.3 × 0.3 µm) fluorescent image stacks (≈ 100 µm depth). To cover the entire CV-PV axes, 2 × 1 tiles were stitched using the image stitching plug-in of Fiji^65^. All images were reconstructed using the software Motion Tracking (http://motiontracking.mpi-cbg.de) as described in^9^. Briefly, for the pre-processing of the 3D images were first denoised using the PURE-LET algorithm^66^ with the maximum number of cycles. Then, a background and shading correction was performed using the tool BaSiC^67^ along the stack. Finally, all channels were aligned to a reference one using the function Correct 3D Drift from Fiji.

### 3D network architecture and training

To generate virtual images of BC and sinusoidal networks from the images of phalloidin, we expanded the deep convolutional neural network toolbox described in^27^ to 3D. We used a modified version of well-stablished network architecture UNet in 3D, with 3 encoder-decoder blocks. For the convolution layers, we used 64 filters of size 3 × 3 × 3. We replace the classification layer with a regression one. We trained an independent network for each structure (i.e. BC and sinusoidal networks). The models were trained using a batch size of 8 with 64 × 64 × 64 image patches as input (phalloidin) and target images (BC or sinusoids staining). The patches were extracted from six Z-Stack images obtained from postnatal day 1, 16, and adult mice, for both networks. We used ~ 36,000 and ~ 25,000 non-overlapping patches for training the networks for the BC and sinusoids, respectively. For the validation, ~ 3600 and ~ 2500 non-overlapping patches were used, respectively. Both networks were trained using the stochastic gradient descent with momentum optimizer (momentum = 0.9, learning rate = 0.05) for 100 epochs using MATLAB2022b.

### 3D image segmentation

The structures were segmented using a maximum entropy local thresholding algorithm. Artifacts generated by the segmentation (holes and tiny isolated objects) were removed by standard morphological operations (opening/closing). The triangulation mesh of the segmented surfaces was generated by the cube marching algorithm and tuned using an active mesh approach. To separate nuclei we used an interactive watershed plug-in in Fiji^65^ and a splitting algorithm in Motion Tracking^9^. In the case of tubular structures, representations of the “medial axis” or “skeleton”, also called central lines, of the networks were generated. Central lines were generated as 3D graphs. HSCs and KCs were reconstructed based on the desmin and F4/80 staining. Finally, the shape of the cell surface (hepatocytes) was determined using an active mesh expansion from the reconstructed nuclei. For details, refer to^9^.

### Morphological spatial analysis of cells and networks (BC and sinusoids)

The cell and nucleus elongation were defined as one minus the ratio of the major to minor elongation axis of the 3D object. Elongation = 1 − (A1/A3), where A1 and A3 correspond to the length of the maximum and minimum elongation axis of the 3D object. The DNA content for each nucleus was computed by assessing the integral intensity within the respective 3D triangle mesh on the original DAPI image. Hepatocyte ploidy was determined by clustering the nuclei based on their DNA content (i.e. 2n, 4n, 8n, 16n)^9^.

For the analysis of the network and stellate and kupffer cells, the volume fraction was estimated as the ratio between the volume occupied by the tissue structure (i.e. networks or cells) and the total tissue volume (excluding the large veins, CV and PV). To determine the radius of the networks, the average distance of each node along the central lines to the triangle mesh was measured.

To address the variation in the morphological parameters across the liver lobule, the CV–PV axis was digitally segmented into ten equally spaced zones, and the mean value of each morphological parameter was computed within each region, as previously described^9,14^.

### Clodronate treatment

KC depletion was achieved by macrophagic suicide^68,69^. Briefly, 100 µl of clodronate liposome suspension (LIPOSOMA, CP-010-010, 5 mg/ml) per 10 gr of the animal was retro-orbitally injected^70^. As a control, mice were injected with a solution of liposomes and PBS. For long-term depletion experiments, mice were injected every 5 days.

### Statistical analysis

Data were analyzed using Prism 9 software. Statistical tests and number of animal were specified in the figure legends.

### Supplementary Information

Supplementary Information 1.

Supplementary Video 1.

## Data Availability

All the imaging data and 3D reconstructions are available from the corresponding author upon request.

## Abbreviations

CV: Central vein
PV: Portal vein
BC: Bile canaliculi
HSC: Hepatic stellate cells
KC: Kupffer cells
DAPI: 4,6-Diamidino-2-phenylindole
CNN: Convolutional neural network
P1: Postnatal day 1
P16: Postnatal day 16
P30: Postnatal day 30

## Code availability

The source code for training and predicting virtual tissue images using CNN is available from https://github.com/hernanmorales-navarrete/TiMiPNet.
