# Supplementary material for: Phenotypic characterization of liver tissue heterogeneity through a next-generation 3D single-cell atlas
Source: Sci Rep. 2024 Feb 3;14:2823. doi: 10.1038/s41598-024-53309-4 (PMC10837128; doi:10.1038/s41598-024-53309-4)
Supplement: Supplementary file 1 — Supplementary Information 1. [file 41598_2024_53309_MOESM1_ESM.pdf]

# Phenotypic characterization of liver tissue heterogeneity through a next-generation 3D single-cell atlas

Dilan Martínez-Torres[1,2*], Valentina Maldonado[1,2*], Cristian Pérez-Gallardo[1,2], Rodrigo Yañez[1,2], Valeria Candia[1,2], Yannis Kalaidzidis[3], Marino Zerial[3], Hernán Morales-Navarrete[4,5**], Fabián Segovia-Miranda[1,2**]

1. Department of Cell Biology, Faculty of Biological Sciences, Universidad de Concepción, Concepción, Chile.
2. Grupo de Procesos en Biología del Desarrollo (GDeP), Faculty of Biological Sciences, Universidad de Concepción, Chile.
3. Max Planck Institute of Molecular Cell Biology and Genetics, Dresden, Germany.
4. Department of Systems Biology of Development, University of Konstanz, Germany.
5. Facultad de Ciencias Técnicas, Universidad Internacional Del Ecuador UIDE, Quito, Ecuador

* These authors contributed equally to this work.
** For correspondence: fabiansegovia@udec.cl (FS), hernan.morales-navarrete@uni-konstanz.de (HM)

**Supp movie 1: 3D single-cell morphometric atlas of liver tissue architecture.**
Central vein (light blue), portal vein (orange), sinusoids (magenta), bile canaliculus (green), nuclei (random colours), hepatocytes (random colours), HSCs (random colours) and KCs (random colours).

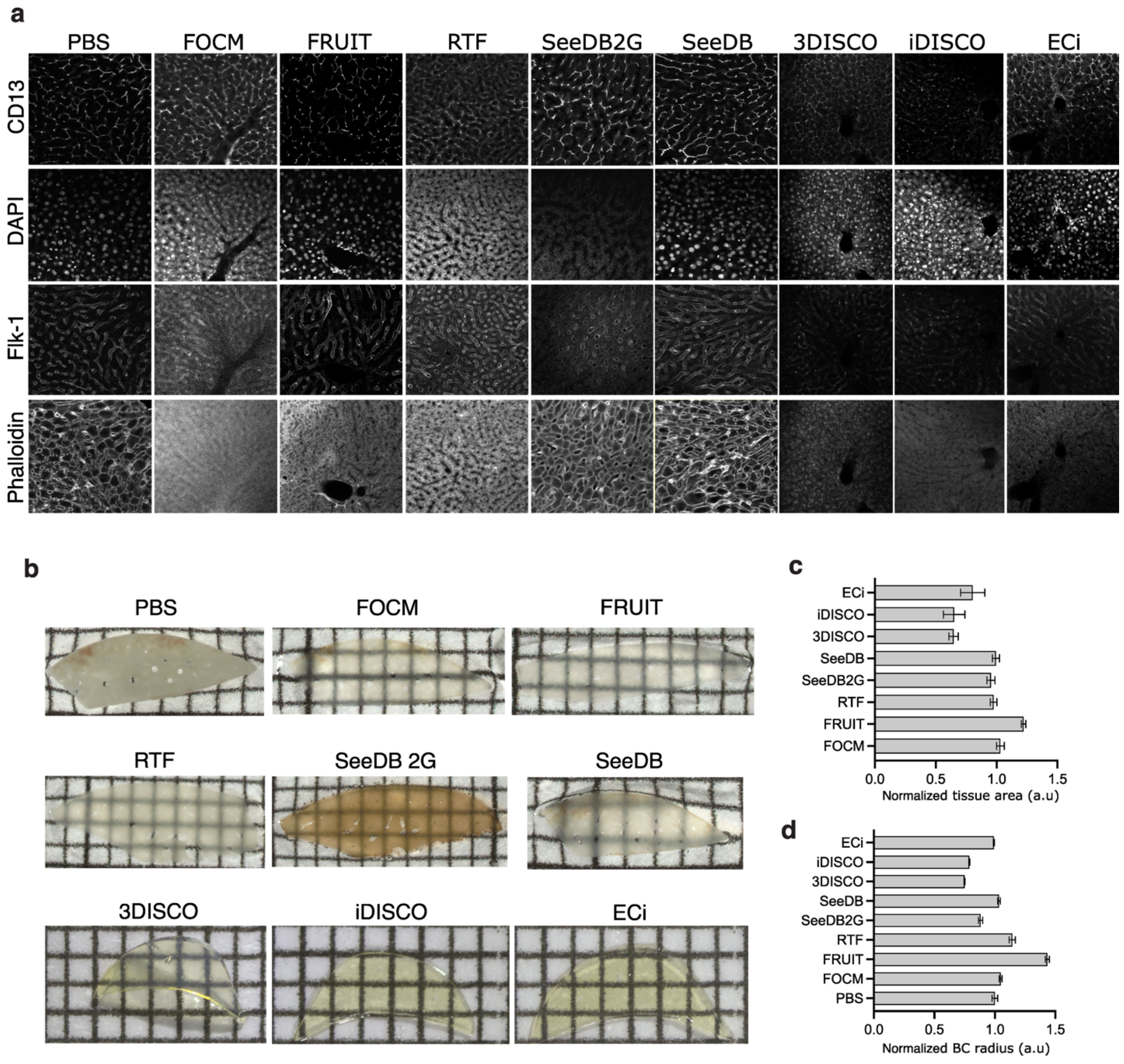


**Supp Fig. 1: Comparison of different optical clearing methods.**

(a) 100µm liver slices were stained with antibodies against CD13 and Flk-1, and the dyes phalloidin and DAPI. Different optical clearings methods were applied and the compatibility with the different markers was qualitatively evaluated. (b) Liver slices before and after the clearing are shown. (c) The outline of the liver slices was drawn in Fiji, the area was calculated and used as a readout of macroscopic tissue deformation. (d) BC was reconstructed with Motion Tracking and the radius was measured. The data were normalized to the PBS condition. The analysis was performed from three adult mice. Quantification represented by mean ± s.e.m.

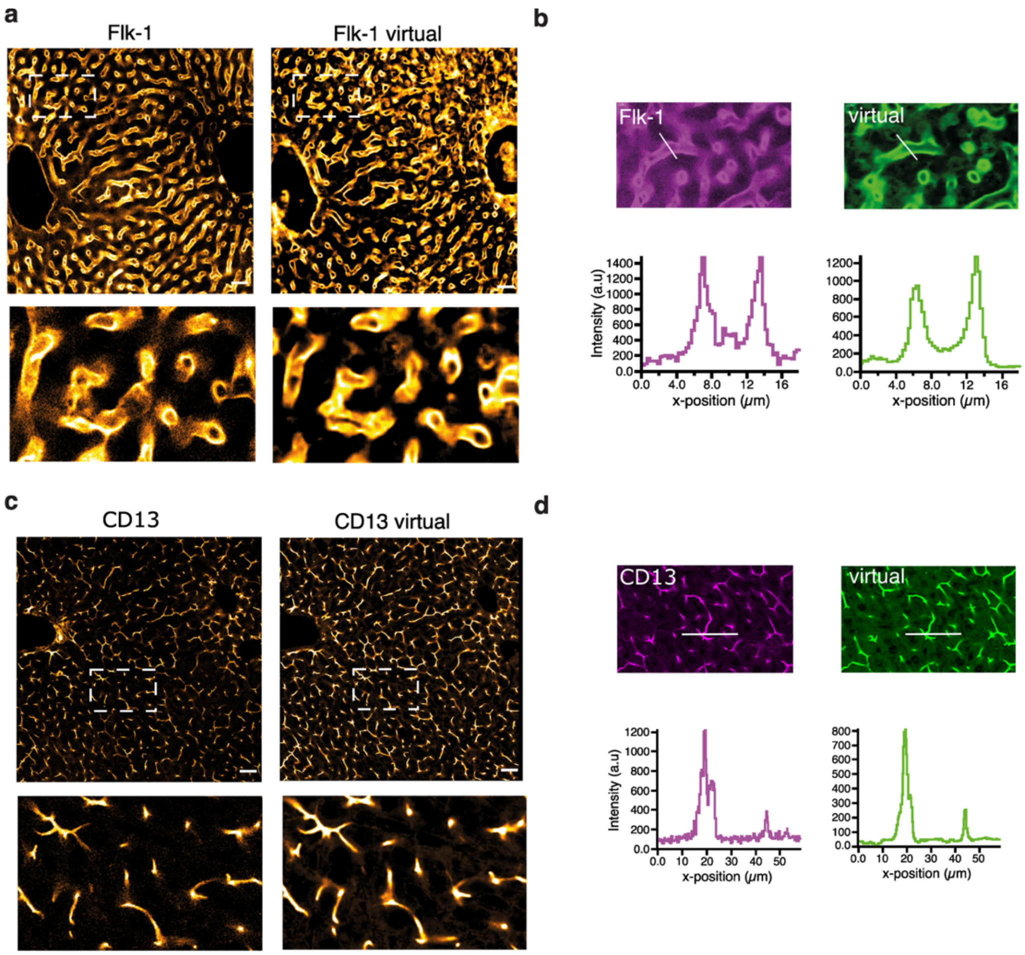


e

| Tissue feature | Sinusoids | Virtual sinusoids |
|---|---|---|
| Total length (m/mm3) | 4.26 | 3.44 |
| Volume fraction (%) | 16.8 | 16.7 |
| Radius (μm) | 2.9 ± 0.88 | 3.2 ± 1.08 |
| Conectivity (fraction) | 0.988 | 0.967 |
| Branch length (μm) | 15.9 ± 7.9 | 15.3 ± 8.01 |
| Junction density (number/μm) | 0.03 | 0.03 |

f

| Tissue feature | BC network | Virtual BC |
|---|---|---|
| Total length (m/mm3) | 6.5 | 6.0 |
| Volume fraction (%) | 3.46 | 4.5 |
| Radius (μm) | 0.91 ± 0.32 | 0.98 ± 0.42 |
| Conectivity (fraction) | 0.97 | 0.97 |
| Branch length (μm) | 10.3 ± 4.38 | 8.4 ± 4.96 |
| Junction density (number/μm) | 0.06 | 0.06 |

**Supp Fig. 2: Artificial neural networks can create images that resemble real staining.**
(a) Representative images of the sinusoids from fixed mouse liver tissue. On the left, liver section stained with Flk-1. On the right, virtual image created with the 3D CNN-based toolbox from the phalloidin staining. Inset showing a magnification of the region highlighted on the upper image. (b) Intensity profiles along the lines drawn on two examples of real marker (magenta) versus virtual (green). (c) Representative images of the BC from fixed mouse liver tissue. On the left, liver section stained with CD13. On the right, virtual image created with the 3D CNN-based toolbox from the phalloidin staining. Inset showing a magnification of the region highlighted on the upper image. (d) Intensity profiles along the lines drawn on two examples of real marker (magenta) versus virtual (green). (e-f) Real and virtual images were 3D reconstructed and some morphometric properties were quantified. The training of the models involved approximately 36000 (for BC) and 25000 (for sinusoids) non-overlapping patches derived from six Z-stacks obtained from postnatal day 1, 16, and adult mice.

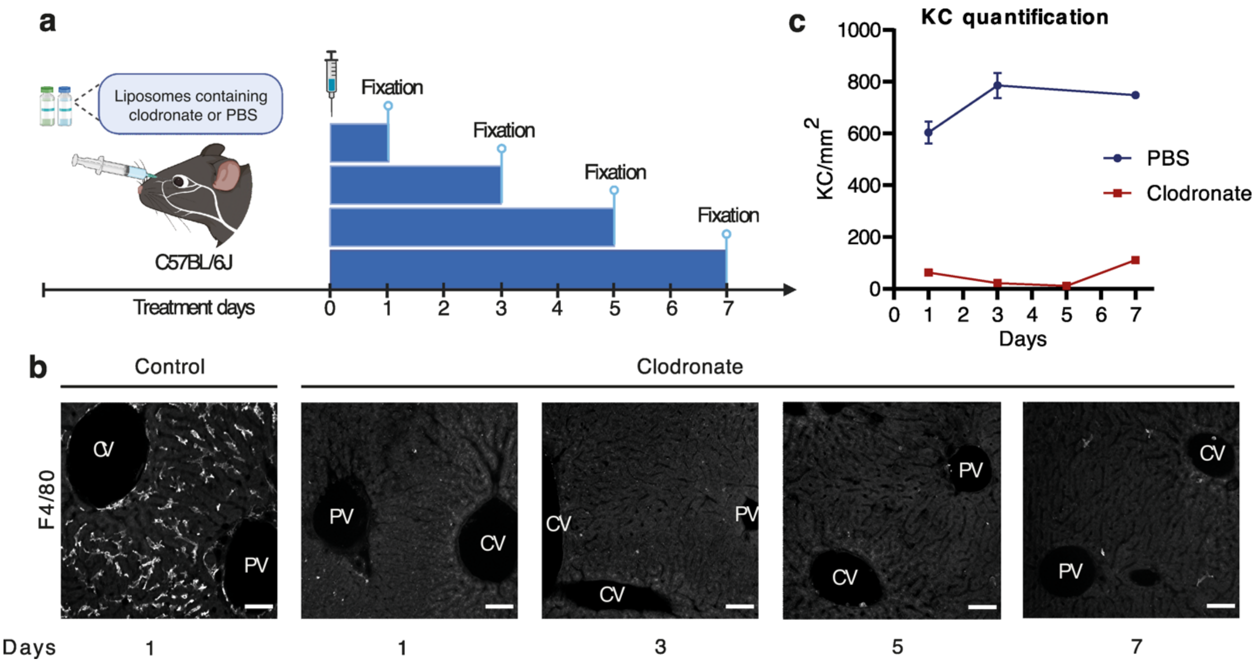


**Supp Fig. 3: Standardization of Kupffer cells depletion with clodronate liposomes.**
(a) Scheme summarizing the depletion experiment. A single clodronate injection was performed and liver samples were obtained after 1, 3, 5 and 7 days. (b) 100 µm thick liver sections were stained with anti-F4/80 antibody. (c) Number of KCs were quantified in Fiji using the images shown in (b) and were compared to control mice injected with PBS. PBS = 3 samples, Clodronate = 3 samples. Quantification represented by mean ± s.e.m.

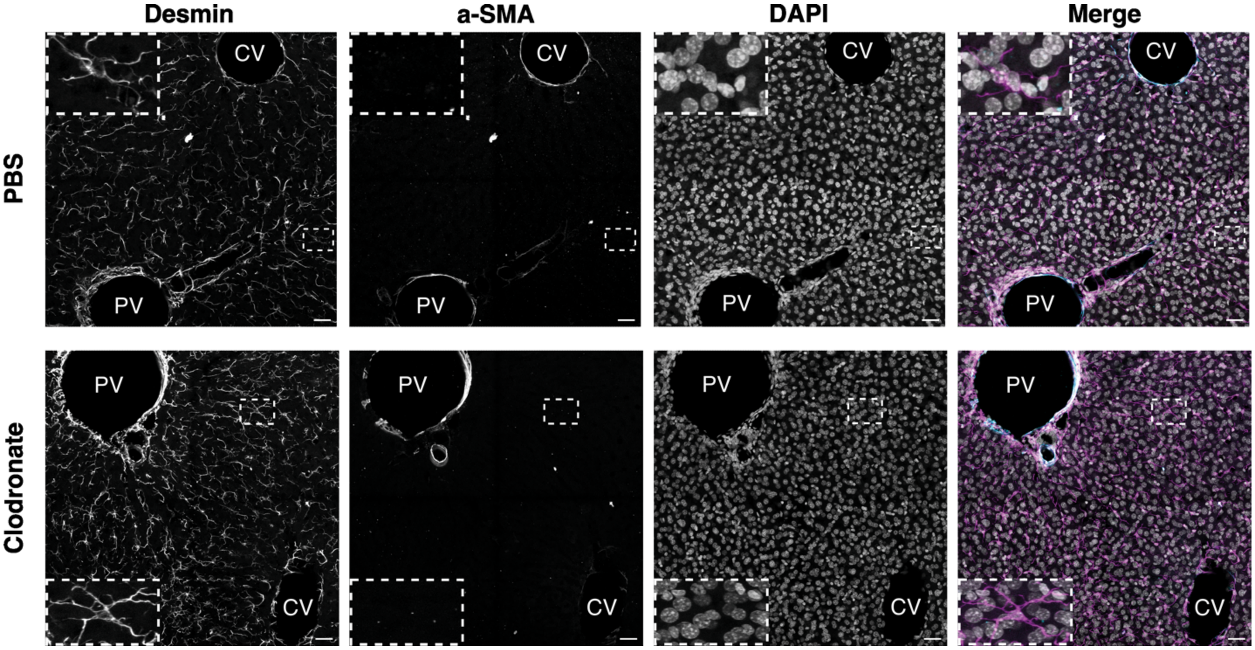


**Supp Fig. 4: HSCs do not get activated upon long-term KCs depletion.**
Liver sections from mice treated with PBS and clodronate were stained with desmin, α-SMA and DAPI. The images represent the maximum projection of a 30 µm z-stack covering an entire CV-PV axis. PBS = 3 samples, Clodronate = 3 samples. Scale bar 30 µm.

**Supp table 01:** Morphometric analysis of BC and sinusoidal networks from early postnatal development to adulthood.

| | Age | P1 | P16 | Adult |
|---|---|---|---|---|
| **Bile canaliculi** | **Total length (m/mm³)** | 4.46 ± 0.5 | 6.17 ± 0.5 | 4.62 ± 0.3 |
| | **Volume fraction (%)** | 6 ± 0.47 | 5.4 ± 0.84 | 4.4 ± 0.59 |
| | **Radius 2D (µm)** | 1.4 ± 0.08 | 1.1 ± 0.04 | 1.1 ± 0.1 |
| | **Connectivity** | 0.84 ± 0.033 | 0.85 ± 0.023 | 0.88 ± 0.008 |
| | **Branch length (µm)** | 8 ± 1.4 | 8.1 ± 0.9 | 8.3 ± 0.5 |
| | **Junction density (number/µm)** | 0.06 ± 0.001 | 0.06 ± 0.001 | 0.06 ± 0.002 |

| | Age | P1 | P16 | Adult |
|---|---|---|---|---|
| **Sinusoids** | **Total length (m/mm³)** | 5.38 ± 0.4 | 6.65 ± 0.4 | 4.75 ± 0.3 |
| | **Volume fraction (%)** | 13.9 ± 0.7 | 27.1 ± 0.88 | 25.2 ± 4.64 |
| | **Radius 2D (µm)** | 2.5 ± 0.04 | 3.2 ± 0.1 | 3.5 ± 0.26 |
| | **Connectivity** | 0.93 ± 0.033 | 0.99 ± 9.5e-5 | 0.99 ± 0.003 |
| | **Branch length (µm)** | 12.8 ± 0.1 | 12.8 ± 0.2 | 14.9 ± 0.6 |
| | **Junction density (number/µm)** | 0.03 ± 0.001 | 0.04 ± 9.4e-5 | 0.04 ± 0.001 |

**Supp table 02:** Morphometric characterization of hepatocytes from early postnatal development to adulthood.

| | Age | P1 | P16 | Adult |
|---|---|---|---|---|
| HEPATOCYTES (GENERAL) | Total number/mm³ | 474336 ± 35903 | 595904 ± 33765 | 235756 ± 31938 |
| | Volume fraction (%) | 58.5 ± 1.4 | 59.6 ± 0.56 | 56.7 ± 2.22 |
| | Cell volume (µm³ ) | 2152 ± 160 | 1731 ± 94 | 4471 ± 525 |
| | Mono-nuclear (%) | 75.8 ± 4.1 | 79.3 ± 7.6 | 63.1 ± 7.5 |
| | Bi-nuclear (%) | 17.3 ± 2.1 | 16.9 ± 4.7 | 34.5 ± 7.1 |
| | Nuclei volume (µm³) | 344 ± 47 | 342 ± 40 | 480 ± 71 |
| | Nuclei elongation | 0.83 ± 0.050 | 0.52 ± 0.061 | 0.4 ± 0.057 |

| | Ploidy | 1 x 2n | | | 1 x 4n | | | 1 x 8n | | |
|---|---|---|---|---|---|---|---|---|---|---|
| | Age | P1 | P16 | Adult | P1 | P16 | Adult | P1 | P16 | Adult |
| HEPATOCYTES (BY PLOIDY) | Percentage of total hepatocytes (%) | 58.9 ± 2.7 | 52.4 ± 1.2 | 27.4 ± 0.8 | 15.5 ± 4.3 | 24.9 ± 5.5 | 27.9 ± 4.4 | 1.4 ± 1.2 | 1.6 ± 0.8 | 7.1 ± 3.7 |
| | Cell volume (µm³) | 1412 ± 82 | 1247 ± 128 | 2175 ± 291 | 1821 ± 329 | 1612 ± 107 | 3756 ± 410 | 2801 ± 286 | 3250 ± 483 | 5949 ± 551 |
| | Elongation | 0.78 ± 0.006 | 0.59 ± 0.025 | 0.65 ± 0.015 | 0.94 ± 0.022 | 0.59 ± 0.026 | 0.57 ± 0.054 | 1.06 ± 0.141 | 0.75 ± 0.065 | 0.52 ± 0.050 |
| | Apical surface (%) | 15.4 ± 0.5 | 18.2 ± 0.8 | 17.8 ± 3.3 | 15.8 ± 0.9 | 18.9 ± 0.5 | 19.8 ± 3.0 | 16.9 ± 3 | 13.2 ± 2.5 | 22.7 ± 2.3 |
| | Basal surface (%) | 23.1 ± 1.2 | 39.1 ± 2.7 | 32.5 ± 2.7 | 23.3 ± 2.5 | 40.6 ± 3.1 | 39.6 ± 4 | 23.8 ± 2.9 | 50.2 ± 7.1 | 42.8 ± 3.8 |
| | Lateral surface (%) | 61.5 ± 1.4 | 42.7 ± 3.6 | 49.7 ± 5.9 | 60.9 ± 3.4 | 40.5 ± 3.6 | 40.6 ± 7 | 59.3 ± 5.8 | 36.6 ± 6.7 | 34.5 ± 6.1 |
| | Number of neighbours | 9 ± 0.24 | 8 ± 0.48 | 8 ± 0.33 | 10 ± 0.69 | 8 ± 0.57 | 9 ± 0.57 | 11 ± 1.4 | 11 ± 0.33 | 11 ± 0.93 |

| Ploidy | 2 x 2n | | | 2 x 4n | | | 2 x 8n | | |
|---|---|---|---|---|---|---|---|---|---|
| Age | P1 | P16 | Adult | P1 | P16 | Adult | P1 | P16 | Adult |
| Percentage of total hepatocytes (%) | 13.6 ± 2.7 | 12.6 ± 5.1 | 15.0 ± 2.8 | 0.5 ± 0.2 | 1.6 ± 0.6 | 15.7 ± 4.5 | 0 ± 0 | 0.1 ± 0.7 | 1.0 ± 0.5 |
| Cell volume (µm³) | 3352 ± 178 | 2586 ± 308 | 4474 ± 529 | 3533 ± 782 | 3220 ± 349 | 7279 ± 596 | 0 ± 0 | 1482 ± 1482 | 9792 ± 603 |
| Elongation | 1.07 ± 0.038 | 0.93 ± 0.030 | 0.65 ± 0.050 | 1.06 ± 0.112 | 1.12 ± 0.038 | 0.67 ± 0.058 | 0 ± 0 | 0.38 ± 0.377 | 0.84 ± 0.144 |
| Apical surface (%) | 19.5 ± 1.7 | 19.7 ± 1.8 | 23.3 ± 3 | 19.3 ± 1.2 | 21.3 ± 1.3 | 22.3 ± 2.1 | 0 ± 0 | 9 ± 9 | 17.4 ± 3.7 |
| Basal surface (%) | 25.6 ± 0.9 | 41.1 ± 2.2 | 38.0 ± 3.6 | 21.9 ± 8.6 | 43.5 ± 2.9 | 44.7 ± 4.7 | 0 ± 0 | 11.5 ± 11.5 | 48.6 ± 7.4 |
| Lateral surface (%) | 54.9 ± 2.6 | 39.2 ± 3.9 | 38.7 ± 6.3 | 58.7 ± 7.5 | 35.3 ± 4.2 | 33 ± 6.6 | 0 ± 0 | 12.9 ± 12.9 | 34.0 ± 10.3 |
| Number of neighbours | 13 ± 0.02 | 10 ± 0.45 | 11 ± 0.68 | 13 ± 1.22 | 12 ± 0.97 | 12 ± 0.49 | 0 ± 0 | 4 ± 4 | 13 ± 0.5 |

**Supp table 03:** Morphometric characterization of non-parenchymal cells from early postnatal development to adulthood.

| | Age | P1 | P16 | Adult |
|---|---|---|---|---|
| **HSC** | **Total number/mm³** | 61449 ± 11523 | 63983 ± 4805 | 44217 ± 7397 |
| | **Volume fraction (%)** | 9.2 ± 1.87 | 9.8 ± 1.84 | 7.3 ± 0.49 |
| | **Cell volume (µm³)** | 1172 ± 64 | 1303 ± 274 | 1578 ± 95 |
| | **Elongation** | 2.73 ± 0.006 | 2.52 ± 0.066 | 2.51 ± 0.118 |
| | **Nuclei volume (µm³)** | 325 ± 31 | 262 ± 45 | 193 ± 52 |
| | **Nuclei elongation** | 1.1 ± 0.03 | 1.18 ± 0.1 | 1.02 ± 0.12 |

| | Age | P1 | P16 | Adult |
|---|---|---|---|---|
| **KC** | **Total number/mm³** | 107528 ± 17238 | 29785 ± 5343 | 18986 ± 3851 |
| | **Volume fraction (%)** | 8.8 ± 1.93 | 7 ± 1.15 | 3.6 ± 0.04 |
| | **Cell volume (µm³)** | 2170 ± 386 | 1934 ± 246 | 1702 ± 86 |
| | **Elongation** | 1.7 ± 0.127 | 2.07 ± 0.120 | 2.34 ± 0.045 |
| | **Nuclei volume (µm³)** | 352 ± 70 | 260 ± 42 | 151 ± 36 |
| | **Nuclei elongation** | 1.01 ± 0.09 | 1.36 ± 0.21 | 1.41 ± 0.3 |

**Supp table 04:** Morphometric analysis of BC and sinusoidal networks in absence of KCs

| | Condition | PBS | Clodronate |
|---|---|---|---|
| Bile canaliculi | Total length (m/mm³) | 4.94 ± 0.1 | 5.37 ± 0.2 |
| | Volume fraction (%) | 6.9 ± 0.72 | 5.66 ± 0.55 |
| | Radius 2D (µm) | 1.4 ± 0.11 | 1.2 ± 0.13 |
| | Connectivity | 0.88 ± 0.018 | 0.91 ± 0.012 |
| | Branch length (µm) | 8.3 ± 0.8 | 8.0 ± 0.3 |
| | Junction density (number/µm) | 0.06 ± 0.001 | 0.06 ± 0.002 |

| | Condition | PBS | Clodronate |
|---|---|---|---|
| Sinusoids | Total length (m/mm³) | 4.41 ± 0.3 | 3.87 ± 0.8 |
| | Volume fraction (%) | 22 ± 1.84 | 23.2 ± 4.76 |
| | Radius 2D (µm) | 3.3 ± 0.16 | 3.7 ± 0.69 |
| | Connectivity | 0.97 ± 0.012 | 0.96 ± 0.029 |
| | Branch length (µm) | 14.3 ± 0.1 | 13.6 ± 0.7 |
| | Junction density (number/µm) | 0.03 ± 0.001 | 0.03 ± 0.004 |

**Supp table 05:** Morphometric characterization of hepatocytes in absence of KCs

| | Condition | PBS | Clodronate |
|---|---|---|---|
| HEPATOCYTES (GENERAL) | Total number/mm³ | 302397 ± 26303 | 318449 ± 44948 |
| | Volume fraction (%) | 57.4 ± 1.86 | 57.7 ± 2.48 |
| | Cell volume (µm³) | 3224 ± 260 | 3321 ± 461 |
| | Mono-nuclear (%) | 69.6 ± 0.8 | 51.4 ± 4.9 |
| | Bi-nuclear (%) | 24.3 ± 1.5 | 39.3 ± 4.9 |
| | Nuclei volume (µm³) | 433 ± 58 | 436 ± 36 |
| | Nuclei elongation | 0.62 ± 0.068 | 0.49 ± 0.049 |

| | Ploidy | 1 x 2n | | 1 x 4n | | 1 x 8n | |
|---|---|---|---|---|---|---|---|
| | Condition | PBS | Clodronate | PBS | Clodronate | PBS | Clodronate |
| HEPATOCYTES (BY PLOIDY) | Percentage of total hepatocytes (%) | 49.9 ± 3.8 | 31.9 ± 6.3 | 18.5 ± 2.8 | 17.1 ± 3.3 | 1.1 ± 0.2 | 2.1 ± 1.4 |
| | Cell volume (µm³) | 1922 ± 86 | 1690 ± 172 | 3123 ± 143 | 2517 ± 161 | 3972 ± 329 | 4388 ± 530 |
| | Elongation | 0.73 ± 0.025 | 0.71 ± 0.035 | 0.68 ± 0.013 | 0.68 ± 0.066 | 0.57 ± 0.038 | 0.63 ± 0.047 |
| | Apical surface (%) | 21.3 ± 3.1 | 17.4 ± 1.7 | 24.5 ± 2.2 | 21.9 ± 1.8 | 28.4 ± 1.7 | 26.5 ± 1.3 |
| | Basal surface (%) | 35.1 ± 2.5 | 37.1 ± 4.1 | 38.7 ± 3.2 | 38.4 ± 6.1 | 35.6 ± 2.7 | 31.0 ± 8.5 |
| | Lateral surface (%) | 43.6 ± 5 | 45.5 ± 3.9 | 36.9 ± 4.6 | 39.7 ± 7.3 | 36.0 ± 2.0 | 42.5 ± 8.9 |
| | Number of neighbours | 8 ± 0.48 | 8 ± 0.78 | 9 ± 0.68 | 9 ± 0.93 | 11 ± 0.48 | 11 ± 1.04 |

| Ploidy | 2 x 2n | | 2 x 4n | | 2 x 8n | |
|---|---|---|---|---|---|---|
| Condition | PBS | Clodronate | PBS | Clodronate | PBS | Clodronate |
| Percentage of total hepatocytes (%) | 15.3 ± 1.9 | 22.9 ± 3.6 | 5.4 ± 2.1 | 11.0 ± 3.1 | 0.4 ± 0.2 | 0.7 ± 0.2 |
| Cell volume (µm³) | 4048 ± 126 | 3296 ± 354 | 6417 ± 504 | 5289 ± 817 | 7471 ± 1438 | 5175 ± 1918 |
| Elongation | 0.8 ± 0.003 | 0.82 ± 0.054 | 0.78 ± 0.056 | 0.79 ± 0.033 | 0.67 ± 0.082 | 0.88 ± 0.374 |
| Apical surface (%) | 25.9 ± 2.5 | 21.9 ± 0.9 | 30.5 ± 0.8 | 24.2 ± 1 | 26.4 ± 2.6 | 25.1 ± 4.2 |
| Basal surface (%) | 39.7 ± 2.8 | 42 ± 5.9 | 41.0 ± 5 | 40.9 ± 7.3 | 41.3 ± 1.5 | 39.9 ± 2.6 |
| Lateral surface (%) | 34.4 ± 4.8 | 36.2 ± 6.1 | 28.5 ± 4.2 | 34.9 ± 6.8 | 32.3 ± 3.6 | 35.1 ± 6.7 |
| Number of neighbours | 11 ± 0.84 | 10 ± 1.07 | 13 ± 1.07 | 12 ± 1.32 | 16 ± 1.11 | 12 ± 2.34 |

**Supp table 06:** Morphometric characterization of HSCs in absence of KCs

| | Condition | PBS | Clodronate |
|---|---|---|---|
| **HSC** | **Total number/mm³** | 40851 ± 11114 | 90995 ± 24403 |
| | **Volume fraction (%)** | 6.5 ± 1.29 | 9.4 ± 2.63 |
| | **Cell volume (µm³)** | 1334 ± 117 | 1263 ± 135 |
| | **Elongation** | 3.02 ± 0.191 | 2.49 ± 0.147 |
| | **Nuclei volume (µm³)** | 307 ± 35 | 308 ± 41 |
| | **Nuclei elongation** | 1.2 ± 0.097 | 0.95 ± 0.100 |

| | Condition | PBS | Clodronate |
|---|---|---|---|
| **KC** | **Total number/mm³** | 23657 ± 1854 | - |
| | **Volume fraction (%)** | 4.8 ± 0.37 | - |
| | **Cell volume (µm³)** | 1753 ± 290 | - |
| | **Elongation** | 2.44 ± 0.079 | - |
| | **Nuclei volume (µm³)** | 323 ± 35 | - |
| | **Nuclei elongation** | 1.2 ± 0.046 | - |

**Supp table 07:** Antibodies, dyes and reagents used for staining and experiments

| Reagent type (species) or resource | Designation | Source or reference | Identifiers | Dilution or concentration | Description |
|---|---|---|---|---|---|
| Primary antibodies | anti-Flk1 (goat polyclonal) | R&D System | AF644 | (1:100) | Sinusoidal marker |
| | anti-CD13 (rat monoclonal) | Novus | NB100-64843 | (1:500) | Canaliculi marker |
| | anti-F4/80 (rat monoclonal) | Abcam | ab6640 | (1:400) | F4/80 + cells marker |
| | anti-Desmin (rabbit polyclonal) | Abcam | ab15200 | (1:200) | Stellate cells marker |
| Secondary antibodies | Donkey anti-Goat Alexa Fluor 647 | Invitrogen | A-21447 | (1:1000) | |
| | Donkey anti-Rat CF 568 | Biotium | 20092 | (1:1000) | |
| | Donkey anti-Rabbit Alexa Fluor Plus 647 | Invitrogen | A32795 | (1:1000) | |
| Small dyes | Phalloidin Alexa Fluor 647 | Invitrogen | A22287 | (1:1000) | Cell border |
| | Phalloidin Alexa Fluor 488 | Invitrogen | A12379 | (1:100) | Cell border |
| | DAPI (4',6-Diamidino-2-Phenylindole, Dihydrochloride) | Invitrogen | D1306 | (1:1000) | Nuclei |
| Reagent | Clodronate and control (PBS) liposome | LIPOSOMA | CP-010-010 | (5mg/ml) | |
